# Optimization of leukocyte-poor platelet-rich plasma preparation: a validation study of leukocyte-poor platelet-rich plasma obtained using different preparer, storage, and activation methods

**DOI:** 10.1186/s40634-019-0190-8

**Published:** 2019-06-03

**Authors:** Naoya Kikuchi, Tomokazu Yoshioka, Yu Taniguchi, Hisashi Sugaya, Norihito Arai, Akihiro Kanamori, Masashi Yamazaki

**Affiliations:** 1 Department of Orthopedic Surgery, Faculty of Medicine, University of Tsukuba, 1-1-1 Tennodai, Tsukuba, Ibaraki 305-8575 Japan; 2 Regenerative Medicine for Musculoskeletal System, Faculty of Medicine, University of Tsukuba, 1-1-1 Tennodai, Tsukuba, Ibaraki 305-8575 Japan

**Keywords:** Platelet-rich plasma, Quality control, Validation study

## Abstract

**Background:**

Alternative methods of platelet-rich plasma (PRP) preparation, storage, and activation that can be stably reproduced are needed to improve PRP production. The purpose of this study was to investigate the effect of the preparer’s experience on the quality of prepared PRP, chronological changes occurring in PRP, and the effect of the activation procedures on the release of several growth factors from PRP, using PRP prepared with the PRGF-Endoret Kit.

**Methods:**

Leukocyte-poor PRP samples from seventeen healthy volunteers were prepared using the PRGF-Endoret Kit and the PRGF IV System Centrifuge. The platelet and leukocyte concentrations were compared based on the preparer’s experience. The concentrations of platelets, hepatocyte growth factor (HGF), platelet-derived growth factor-BB (PDGF-BB), and insulin-like growth factor-1 (IGF-1) were determined at 0 and 60 min after PRP preparation, and compared. Concentrations of the above growth factors from PRP activated by freeze–thaw cycling and by calcium chloride (CaCl_2_) were also compared.

**Results:**

No significant difference was observed in the platelet concentrations and leukocyte contamination rates, based on the preparer’s experience. At 60 min after PRP preparation, the platelet concentration decreased significantly, while the HGF, PDGF-BB, and IGF-1 concentrations remained unchanged. Activation with CaCl_2_ resulted in a significant increase in the PDGF-BB levels, although the HGF and IGF-1 concentrations remained unchanged.

**Conclusions:**

The results of this study show that leukocyte-poor PRP prepared using the PRGF-Endoret Kit did not result in any qualitative difference that depended on the experience of the preparer. However, PRP preparation required standardization in terms of the time of blood count measurement. Growth factor concentrations in PRP differed according to the platelet-activation method used.

## Background

Platelet-rich plasma (PRP) has been defined as “a volume of autologous plasma that has a platelet concentration above baseline” (Marx, 2001). Essentially, PRP therapy for treating tissue damage involves the complex activities of cell-adhesion molecules, glycoproteins, and various growth factors found in concentrated platelet alpha-granules and plasma, which collectively maintain physiological balance within the body (Alsousou et al., 2009; Foster et al., 2009).

PRP variability depends on 2 main factors (Chahla et al., 2017). One factor is the patient’s characteristics, such as his/her age, sex, and consumption of non-steroidal anti-inflammatory drugs (Xiong et al., 2018; Taniguchi et al., 2019; Schippinger et al., 2015). The other factor involved in PRP variability is the PRP-preparation methods used, such as the sample volume, centrifugation step(s), storage conditions, and means of PRP activation (Foster et al. 2009; Lopez-Vidriero et al., 2010).

Many PRP-purification systems are commercially available (Wasterlain et al., 2012), and prepared PRP varies in terms of the platelet concentration, degree of leukocyte enrichment, and concentration of growth factors (Dhurat & Sukesh, 2014). We prepared PRP using the PRGF-Endoret Kit (BTI Biotechnology Institute, Vitoria-Gasteiz, Spain) and PRGF system IV centrifuge, and used the PRP obtained for basic and clinical research (Aoto et al., 2014; Taniguchi et al., 2018; Taniguchi et al., 2019; Yoshioka et al., 2013). Using this system, preparation is conducted with a single centrifugal separation (2100 rpm, 8 min), followed by manual separation using a dedicated pipette, which finally results in the isolation of leukocyte-poor, PRP (LP-PRP). To accurately assess the outcomes of future basic and clinical studies, we considered the necessity of a PRP preparation, storage, and activation method that can be stably reproduced.

PRP preparation, using the PRGF-Endoret system, is performed according to the following procedure: 1. a fixed amount of blood is collected, 2. the whole blood components are centrifugally separated using an automated centrifuge, and 3. the PRP is manually extracted with an aspirator. Because PRP extraction is performed manually, its quality may be compromised due to human error or inefficiency. However, we have no data regarding the accuracy of PRP extraction.

Because it is difficult to perform immediate measurements of growth factor concentrations in normal clinical practice, the PRP quality is assesses by counting blood cells and measuring platelet and leukocyte concentrations. However, when PRP is left to stand, the platelets precipitate, clump, and become activated (Kaplan et al., 1979), which may affect the subsequent platelet concentration and growth factor measurements. Thus, if blood cell counts are not performed at the same fixed time, then the PRP-quality assessments may differ. Some studies (Marx, 2001; Su et al., 2008) have reported chronological changes in growth factors released from platelets; however, no reports have described chronological changes occurring in PRP prepared using the PRGF-Endoret Kit.

It is important to perform a quantitative assessment of growth factor concentrations while assessing PRP quality. Platelets contain alpha granules, which carry various cargo proteins, including growth factors. Activation of growth factors implies their release from platelets, which are achieved by degranulation of the alpha granules (Zimmermann et al., 2003). There are two methods for activation: a mechanical method (Lacoste et al., 2003), which involves thawing frozen platelets, and a chemical method (Pietrzak & Eppley, 2005), which involves the addition of calcium chloride (CaCl_2_). Some findings have suggested that different methods of platelet activation result in different levels of growth factors (Hamilton et al., 2015). No study has been conducted to compare the differences in growth factor concentrations resulting from these two methods when using the PRGF-Endoret system.

PRP variability depends on many factors. Therefore, although it was not possible to fully standardize PRP in this study, we performed three experiments with PRPs prepared using the PRGF-Endoret system to address three important points. The aim of this study was to investigate the effect of the preparer’s experience on the quality of the resulting PRP, chronological changes occurring in PRP, and the effects of the activation procedures on the release of several growth factors from PRP samples prepared using the PRGF-Endoret system. We hypothesized that an experienced person would prepare PRP samples with higher platelet concentrations and lower leukocyte-contamination rates. We also hypothesized that as the platelet concentration was decreased, the growth factor concentration would increase when PRP samples were left standing. In addition, we hypothesized that PRP samples activated with CaCl_2_ would release more growth factors than PRP samples activated with freeze–thaw cycling alone.

## Methods

### Subjects

Seventeen healthy volunteers (mean age: 26.3 years) were enrolled in this study; their ages ranged between 23 and 31 years. The study protocol was approved by our Institutional Committee on Human Research. Specimens were collected after obtaining informed consent from the donors and with the approval of the ethics committee of the University of Tsukuba.

### LP-PRP preparation

Peripheral blood (72 ml) was obtained from the median cubital vein using 22-gauge needles by one researcher, and was placed in eight 9-ml sterile extraction tubes containing 3.8% trisodium citrate. Using the PRGF-Endoret system, the blood was centrifuged once at 2100 rpm for 8 min at room temperature to separate the different phases. A line was drawn at 5 mm above the buffy-coat layer in the separated blood, and the layer above this line was divided into two portions. The upper portion was designated as platelet-poor plasma (PPP) and the lower portion was designated as the LP-PRP. Four different researchers removed the PPP by aspiration and then extracted 2 ml of LP-PRP from each blood sample. Each researcher extracted a total of 4 ml LP-PRP from the two tubes, 3 ml of which was stored and activated differently (described below), and the remaining 1 ml was used for hematological analysis.

### Experimental design

In this study, we performed three experiments with the same volunteer specimens as described below.

#### Experiment 1: studying the effect of the preparer’s experience on the quality of the resultant PRP

Four orthopedic surgeons were divided into two groups as follows: group E consisted of two orthopedic surgeons with clinical experience in preparing PRP samples, and group NE consisted of two orthopedic surgeons without such experience. Those in group NE had watched an instructive DVD produced by the BTI Biotechnology Institute only once beforehand.

The prepared PRP samples were classified as per the PAW Classification system (DeLong et al., 2012), based on the platelet count, activation method, and white blood cell (leukocyte) status (i.e., presence or absence). PRP, produced using the PRGF-Endoret system, was classified as P2-B-β using the PAW classification system. PRP samples prepared by the surgeons in groups E and NE were compared in terms of their blood platelet concentrations (number of PRP platelets/number of whole blood platelets), leukocyte-contamination rates (number of PRP leukocytes/number of whole blood leukocytes), and growth factor concentrations. To assess the learning curve, we also compared the platelet concentrations and leukocyte contents in groups E and NE at three stages during the preparation procedure (initial stage: 5 samples, mid-stage: 6 samples, final stage: 6 samples), as described previously (Khan et al., 2014). We also investigated correlations between platelet concentrations and growth factor concentrations in the PRP samples.

#### Experiment 2: chronological changes in the PRP samples

We utilized PRP samples prepared by a preparer from group E in Experiment 1. Blood cell counts were measured after PRP preparation. The samples were divided into two groups: one group whose blood cell counts were measured immediately and then stored frozen at − 80 °C (immediate group) and another group whose blood cell counts were measured at 60 min and then stored frozen at − 80 °C (60-min group). The platelet and growth factor (HGF, PDGF-BB, and IGF-1) concentrations in both groups were subsequently compared.

#### Experiment 3: effect of the activation procedures on the PRP samples

The PRP samples, prepared by a preparer in group E (as per experiment 1), were either stored frozen at − 80 °C without undergoing activation (F-T group) or activated with CaCl_2_ prior to being stored frozen at − 80 °C (CaCl_2_ group). Subsequently, the concentrations of growth factors (HGF, PDGF-BB, and IGF-1) were compared. In the CaCl_2_ group, 50 μl of 10% CaCl_2_ was added to 1.0 ml of each PRP studied, incubated for 60 min at 37 °C, and centrifuged at 1000×*g* for 20 min at 4 °C. The resulting supernatant was then aspirated and stored frozen at − 80 °C.

### Hematological analysis

The white blood cell (WBC) and platelet counts of the whole-blood samples, and the LP-PRP levels were determined using an automated cell count analyzer (Sysmex KX-21 N, Kobe, Japan).

### Measurement of growth factors

A single freeze–thaw cycle was used to induce platelet activation and growth factor release. The samples were thawed and centrifuged for 10 min at 10,000 rpm, and the supernatants were examined. Three growth factors (HGF, IGF-1, and PDGF-BB) were measured using a commercially available enzyme-linked immunosorbent assay kit (R&D systems, Minneapolis, MN), according to the manufacturer’s instructions.

### Statistical analysis

All results were expressed as the mean **±** SD. The Mann–Whitney U test was used to compare platelet concentrations, leukocyte-contamination rates, and growth factor concentrations between group E and group NE. One-way ANOVA and Kruskal–Wallis testing were used to assess the learning curve. Spearman’s rank correlation analysis was applied to determine correlations between platelet and growth factor concentrations in PRP samples. A paired t-test was used to compare the concentrations of platelets and growth factors between the immediate group and the 60-min group, and to compare platelet concentrations between the F-T group and the CaCl_2_ group.

A *P*-value of < 0.05 was considered to indicate a statistically significant difference. All statistical analyses were performed using SPSS Statistics software, version 21.0 (International Business Machines Co., New York City, NY).

## Results

### Subject profiles

Analysis of whole blood samples indicated that the mean leukocyte concentration was 60.8 ± 11.0 × 10^2^/μl and the mean platelet concentration was 20.8 ± 3.5 × 10^4^/μl.

#### Experiment 1: effect of the preparer’s experience on the quality of the resulting PRP samples

**Fig. 1:**
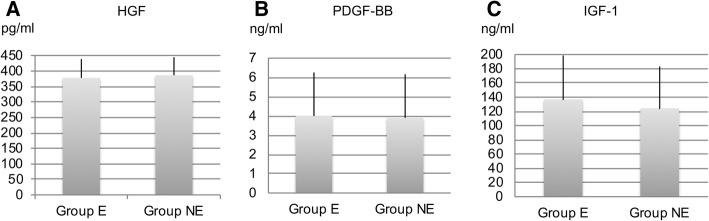
Concentrations of (**a**) HGF, (**b**) PDGF-BB, and (**c**) IGF-1 in the PRP samples. No significant differences in the concentrations of these growth factors were observed between the E and NE groups (*p* > 0.05). Data are presented as the mean ± SD

**Fig. 2:**
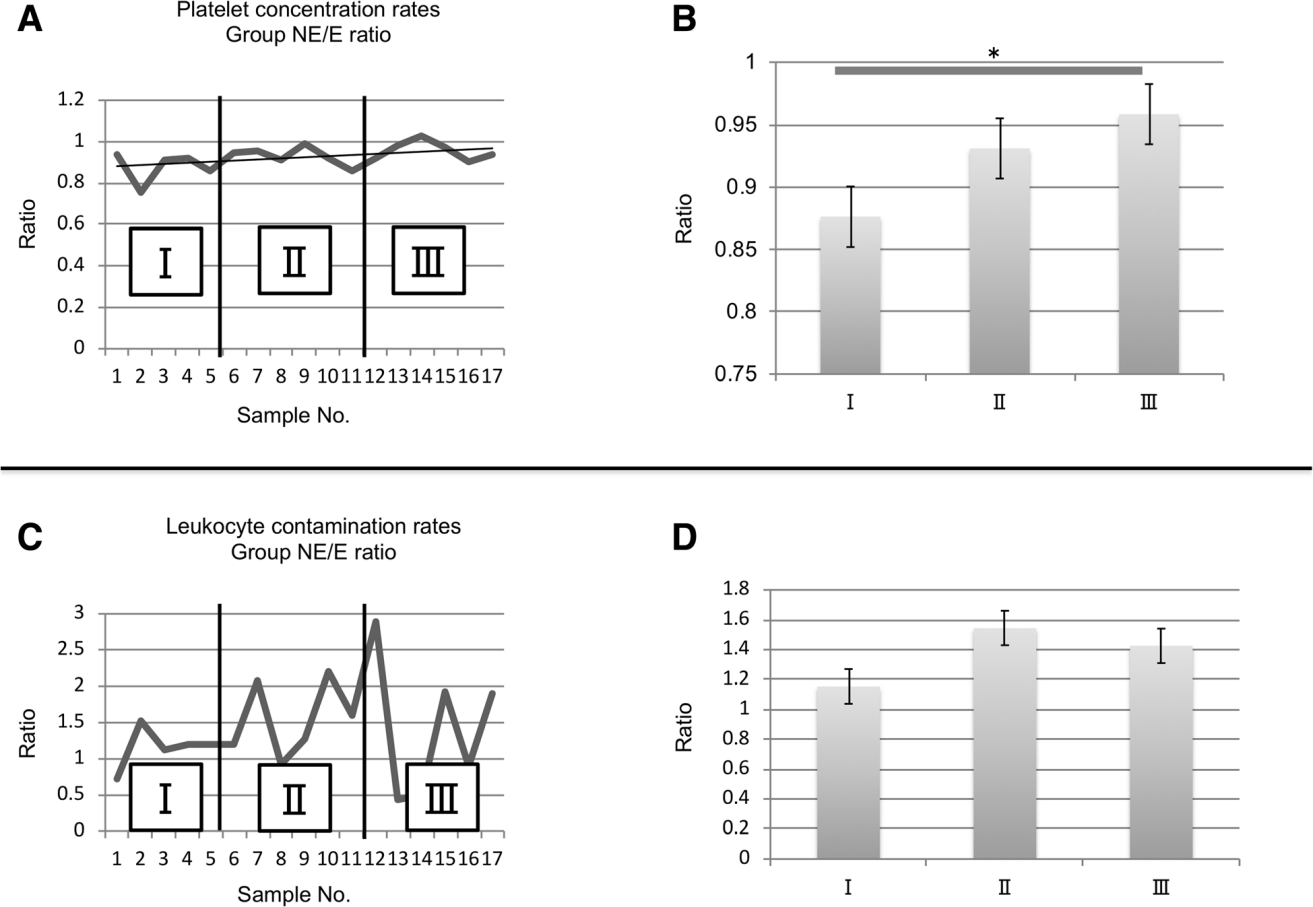
**a** Platelet concentration rates in Group E and NE ratio were divided into three stages in the preparation procedure (stage I: 5 samples, stage II: 6 samples, stage III: 6 samples). **b** A significant difference was shown between stage I and stage III in platelet concentration rates of Group NE/E ratios (*p* < 0.05). **c** Leukocyte contamination rates in Group E and NE ratio were divided into three stages in the preparation procedure (stage I: 5 samples, stage II: 6 samples, stage III: 6 samples). **d** Leukocyte contamination rates of Group NE/E ratio showed no significant differences at any of the three stages

**Fig. 3:**
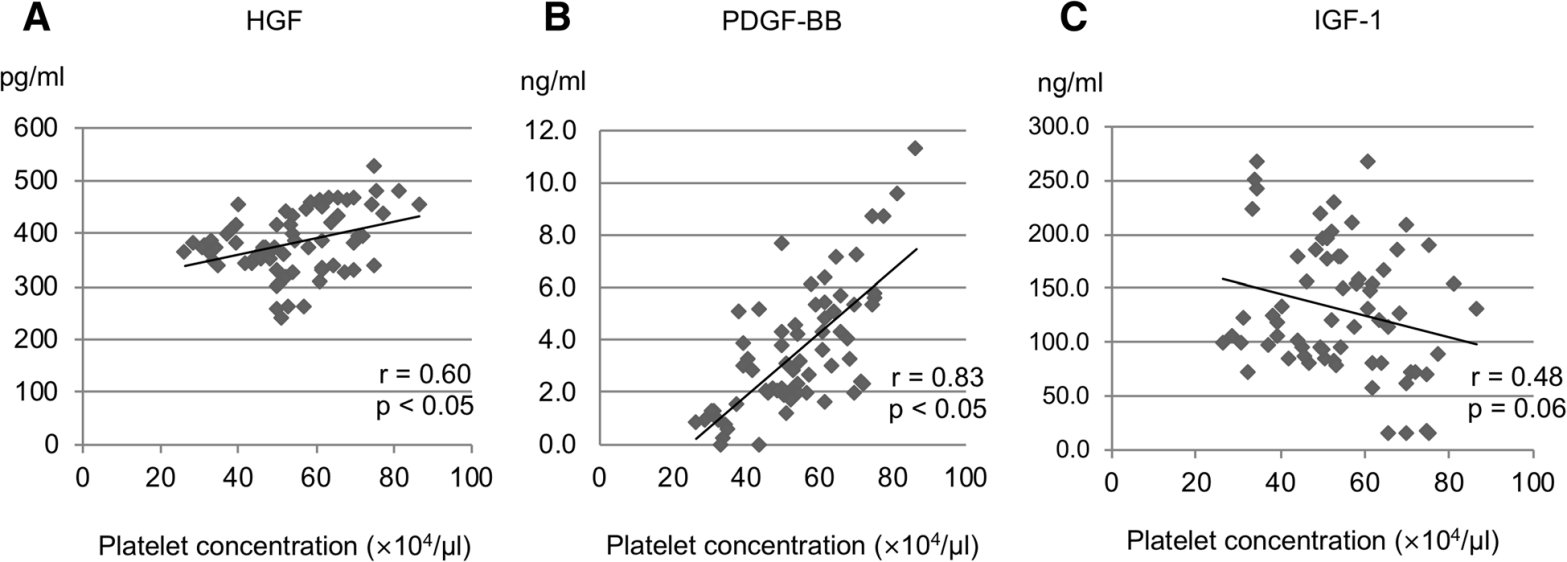
Correlations between the platelet concentration and (**a**) HGF, (**b**) PDGF-BB, and (**c**) IGF-1 levels. A significant positive correlation was found between the HGF and platelet concentrations (r = 0.60, *p* < 0.05), and between the PDGF-BB and platelet concentrations (r = 0.83, *p* < 0.05)

All PRP samples in groups E and NE were considered P2-B-β samples, based on the PAW classification system. The platelet and leukocyte-contamination rates were 2.70-fold and 1.12%, respectively, in group E, and 2.50-fold and 1.40%, respectively in group NE, indicating that no significant difference occurred. The growth factor concentrations were as follows: HGF, 377.7 ± 60.8 pg/ml in group E and 386.3 ± 58.2 pg/ml in group NE; PDGF-BB, 3.99 ± 2.26 ng/ml in group E and 3.90 ± 2.26 ng/ml in group NE; and IGF-1, 136.9 ± 61.7 ng/ml in group E and 124.5 ± 58.7 ng/ml in group NE, thus indicating that no significant differences occurred (Fig. 1). The ratios of platelet concentrations in group NE to those in Group E were 0.88 at the initial stage and 0.96 at the final stage, suggesting a significant difference. The ratios of leukocyte contamination rate in group NE to those in group E showed no significant differences at any of the three stages (Fig. 2). A significant positive correlation was observed between HGF and platelet concentrations (r = 0.60, *p* = 0.002), as well as between PDGF-BB and platelet concentrations (r = 0.83, *p* < 0.0001). However, no significant correlation was found between IGF-1 and platelet concentrations (r = − 0.48, *p* = 0.06) (Fig. 3).

#### Experiment 2: chronological changes in PRP samples

**Fig. 4:**
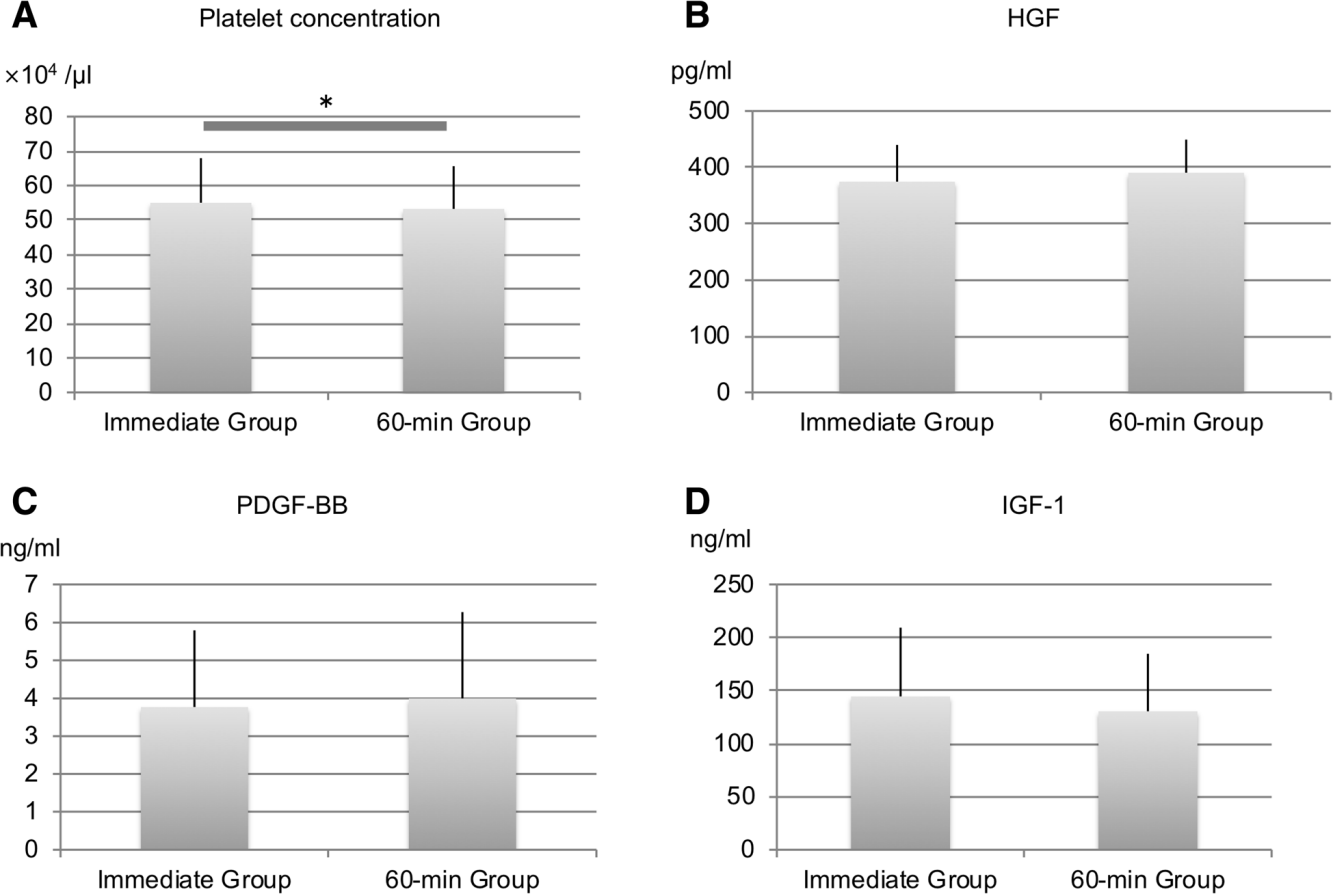
Concentrations of (**a**) platelets, (**b**) HGF, (**c**) PDGF-BB, and (**d**) IGF-1 in the PRP samples. Platelet concentrations were higher in the immediate group (*p* < 0.05). No significant difference was observed in the concentrations of the three growth factors between the E and NE groups (*p* > 0.05)

The platelet concentration was 55.0 ± 12.9 × 10^4^/μl in the immediate group and 53.2 ± 12.4 × 10^4^/μl in the 60-min group, indicating that a statistically significant difference occurred. Growth factor concentrations were as follows: HGF, 374.2 ± 65.4 pg/ml in the immediate group and 390.1 ± 59.0 pg/ml in the 60-min group; PDGF-BB, 3.77 ± 2.02 ng/ml in the immediate group and 3.99 ± 2.27 ng/ml in the 60-min group; and IGF-1, 144.6 ± 64.6 ng/ml in the immediate group and 130.5 ± 54.0 ng/ml in the 60-min group, indicating that no significant differences occurred (Fig. 4).

#### Experiment 3: effect of the activation procedures on the PRP samples

**Fig. 5:**
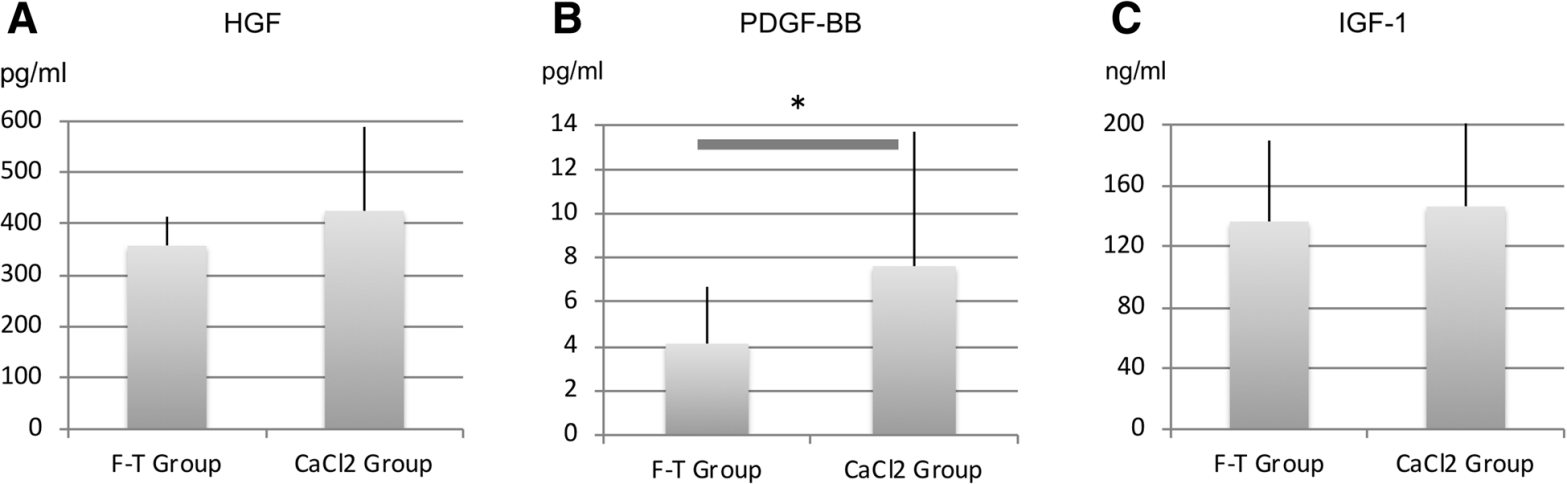
Concentrations of (**a**) HGF, (**b**) PDGF-BB, and (**c**) IGF-1 in the PRP samples. PDGF-BB levels were significant higher in the CaCl_2_ group (*p* < 0.05). No significant differences were observed in the concentrations of the other growth factors between the E and NE groups (*p* > 0.05)

The growth factor concentrations were as follows: HGF, 358.1 ± 56.2 pg/ml in the F-T group and 425.7 ± 163.4 pg/ml in the CaCl_2_ group; PDGF-BB, 4.13 ± 2.56 ng/ml in the F-T group and 7.62 ± 6.08 ng/ml in the CaCl_2_ group; IGF-1, 136.4 ± 53.1 ng/ml in the F-T group and 146.5 ± 56.1 ng/ml in the CaCl_2_ group; a significant difference was observed only for the PDGF-BB concentrations (Fig. 5).

## Discussion

This study showed three major results. First, prior experience in PRP preparation had no effect on the quality of the resulting PRP samples; there was a learning curve, and the reproducibility of same-quality PRP increased with practice. Second, at 60 min after PRP preparation, changes began to occur in the platelet concentrations. Finally, CaCl_2_-activated PRP samples that were stored frozen showed higher growth factor concentrations than freeze–thaw-activated PRP samples.

Some reports have shown a comparison of PRP compositions prepared using various PRP systems including, both manual and automated systems, although those studies were not focused on the compositions of PRP samples prepared by different preparers (Kushida et al., 2014; Magalon et al., 2014; Castillo et al., 2011). Our study was the first to investigate the quality of PRP samples using the PRGF-Endoret Kit, based on the preparer’s experience. No statistically significant differences were observed between the two groups with differential experience levels, in terms of the platelet concentrations and leukocyte-contamination rates, and both groups prepared PRP samples with the same quality, as per PAW classification. Similarly, no statistically significant differences were found in terms of the growth factor concentrations. In contrast to our hypothesis, we found that experience in preparing PRP had no effect on the quality of the prepared PRP.

**Fig. 6:**
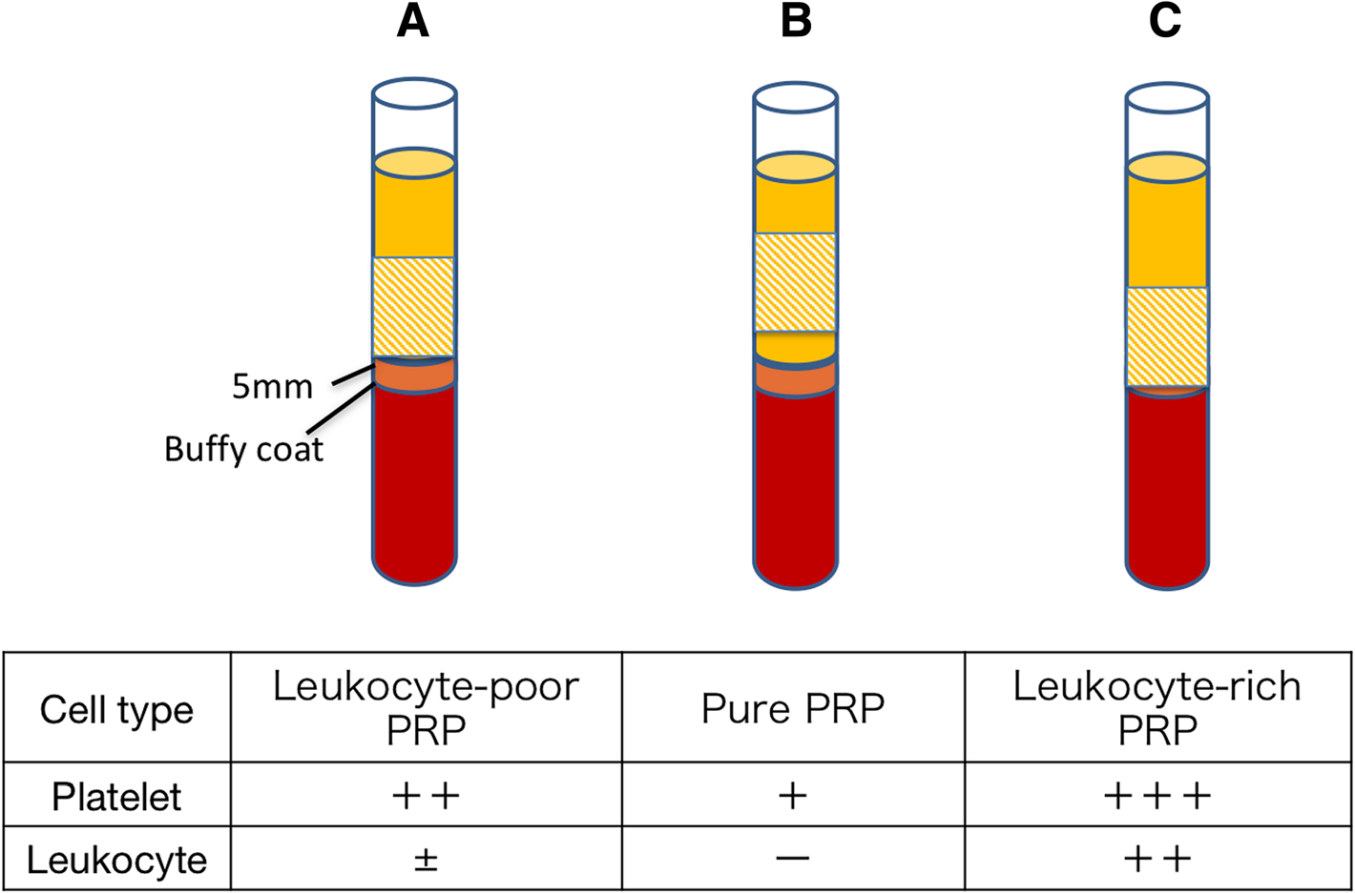
Scheme of the the centrifuged blood with PRGF-Endoret protocol. A line was drawn 5 mm above the buffy-coat layer in the separated blood, and the layer above this line was divided into two. The upper portion was designated as PPP, and the lower portion was designated as PRP. **a** The target layer (shaded area) of the PRP system, containing LP-PRP. **b** When aspirating from above the target layer (shaded area), the platelet concentration obtained was relatively low, without contaminating leukocytes, representing purified PRP. **c** Aspirating from layers below the target layer (shaded area), platelet concentration increased, though leukocytes were retained, which was leukocyte-rich PRP

Broadly speaking, centrifuged whole blood was divided vertically into (from top down) a plasma layer, a leukocyte layer, and a red blood cell layer. The “buffy coat” consisted of the leukocyte layer, whereas the plasma layer immediately above that layer contained a large number of platelets. Platelet concentrations in the plasma layer increased with distance, and platelets were also present in the buffy coat. Our objective was to use the kit to prepare LP-PRP samples with a high concentration of platelets, while excluding leukocytes. Therefore, the PRP samples were manually aspirated from the region up to 5 mm immediately above the buffy coat (Fig. 6a). When each PRP was aspirated from above the target layer, the leukocytes were not included, the platelet concentration was lower and represented purified PRP (Fig. 6b). Conversely, when aspiration proceeded from below the target layer, the platelet concentration increased, resulting in a higher leukocyte concentration and the recovery of leukocyte-rich PRP (Fig. 6c).

When aspirating from layer above the target layer (shaded area), the platelet concentration obtained was lower and leukocytes were not included, resulting in the recovery of purified PRP. When aspirating from layers below the target layer (shaded area), the platelet concentration obtained was higher and leukocytes were retained, resulting in the recovery of leukocyte-rich PRP.

To investigate the associated learning curve, we divided the preparation procedure into three stages (Khan et al., 2014). A statistically significant difference observed in the ratio of the platelet concentration rate in group NE to that in group E suggested that proficiency improved during the mid-stage (6–11 samples). A possible reason explaining this outcome is that the preparers obtained feedback regarding the results of the previous PRP preparation, and further improved their skill in extraction. No statistically significant difference was observed in the leukocyte-contamination rate in group NE versus that in group E. The specific leukocyte content was 11–369/μl, which stabilized at 100/μl or below, in the mid-stage. Leukocyte classification using the PAW classification system divided the total leukocyte count into either higher or lower than that in peripheral blood samples and further divided the neutrophil count into either higher or lower than that in peripheral blood. Considering that the leukocyte concentration of whole blood was 60.8 ± 11.0 × 10^2^/μl, the leukocyte count of the manually extracted PRP was lower, regardless of the preparer. In fact, training made it possible to prepare PRP samples with higher platelet concentrations and standardized training in the procedure is recommended, although prior experience in PRP preparation had no effect on the quality of the resulting PRP samples.

When the PRP samples were allowed to stand for 60 min after preparation, the platelet concentration decreased significantly. Although the HGF and PDGF-BB concentrations did not show significant differences, they were maintained high levels. Consistent with our hypothesis, when the PRP was allowed to stand, the platelet concentrations decreased and growth factor concentrations increased.

The alpha-granules within platelets contain growth factors; thus, when PRP samples are left standing, platelets naturally precipitate, clump, and get activated, thereby releasing the growth factors contained within (Kaplan et al., 1979). During this experiment, the platelet count decreased 60 min after PRP preparation. In experiment 1, the HGF and PDGF-BB concentration correlated positively with the platelet count. In experiment 2, no statistically significant difference was observed in these growth factor concentrations between the 60-min group and the immediate group, although the growth factor concentrations were higher in the 60-min group. These results suggest that the platelets in the PRP samples may have precipitated, clumped, and become activated after 60 min, hence leading to the release of growth factors.

When investigating chronological changes in PRP growth factor concentrations on the surfaces of implants used in dental treatments, Sanchez et al. (2013) found that differences in the activation method and type of growth factor led to differences in these dynamics. Durante et al. (2013) reported chronological changes occurring in eight types of growth factors released from platelets at 5, 10, 20, 30, and 60 min after activation via CaCl_2_. The concentrations of some growth factors increased, whereas those of others decreased, and even in the former case, the timings varied from case to case. Among the growth factors investigated in this experiment, the PDGF-BB concentration increased significantly within 10 min, but no increase was observed thereafter. IGF-1 showed no statistically significant difference in concentration at any time point. Su et al. (2008) reported that IGF-1 concentrations did not differ significantly at 5, 60, 120, and 300 min after activation.

In this study, the differences in the absolute values of the platelet concentrations were very small, but these differences were statistically significant. A detailed description of the PRP composition is required (Chahla et al., 2017) and to determine whether a clinical outcome is attributed to the PRP composition, it is vital to know the platelet concentration in the blood used to generate PRP and in the PRP sample administered to the patient.

However, both studies investigated platelet apheresis with high platelet concentrations, but not with PRP. In addition, since activation was achieved through the use of CaCl_2_, it is impossible to make a direct comparison with the present study. It is necessary to understand growth factor dynamics in platelets, since they differ depending on the specific growth factors studied.

Our results suggest that higher concentrations of growth factors are obtained by storing frozen PRP samples after CaCl_2_ activation than by freeze–thaw cycling alone. Consistent with our hypothesis, the CaCl_2_-activated PRP samples released more growth factors than the freeze–thaw-activated PRP samples.

The freeze–thaw method causes physical destruction of platelets, followed by the release of growth factors from the platelets. CaCl_2_ reacts with the plasma within PRP and produces autologous thrombin, which subsequently activates the platelets and releases growth factors (Whitman et al., 1997). Because the mechanisms of activation differ, the growth factor concentrations obtained from these different methods may be considered to differ.

Hamilton and Best (2011) reported the concentrations of four types of growth factors within PRP that were activated using freeze–thaw and CaCl_2_ activation methods. However, of the three growth factors that we investigated, they measured only HGF and IGF-1. Their study showed that activation with CaCl_2_ resulted in significantly increased IGF-1 concentrations and significantly reduced HGF concentrations. However, the PRP samples they used were prepared using a different PRP-preparation system. Thus, since the preparation method differed from that used in this study, a direct comparison of both studies is not possible.

In addition, the timing of growth factor release also varies according to the activation method utilized. The use of CaCl_2_ for activation accelerates the coagulation response of PRP, thereby speeding up the release of growth factors. As a result, in clinical settings, growth factor levels functioning in target tissues may be reduced before administering PRP.

Activation using CaCl_2_ accelerates gelling of the fibrin bridge, which facilitates PRP handling when stored in tissues, but makes injection difficult when using small-bore needles (Dhurat & Sukesh, 2014) Thus, in clinical settings, the activation method should be selected according to the target tissue and treatment objective.

In this study, we analyzed PDGF-BB, HGF, and IGF-1 levels in three experiments. These growth factors were studied because PDGF-BB is contained in platelets, and HGF and IGF-1 are contained in plasma but not platelets. Moreover, PDGF-BB and HGF levels correlated with the platelet count in our previous study, whereas IGF-1 levels did not (Taniguchi et al., 2019). We analyzed growth factors that show various dynamics and have different origins. PDGF-BB is platelet-derived growth factor, and we investigated whether its levels positively correlated with platelet counts. It was previously reported that the levels of other platelet-derived growth factors were positive correlated with platelet counts (Anitua et al., 2009). In this study, HGF levels derived from plasma positively correlated with platelet counts, whereas IGF-1 (also derived from plasma) did not. These two growth factors may not necessarily correlate only with platelet counts, and it is necessary to quantify these growth factors when evaluating the clinical use of PRP.

Other factors, such as the temperature and time of drawing blood, should be considered. This study was conducted in the same room under a controlled temperature. Previously, it was reported that significant diurnal variations in platelet counts and growth factor levels were not observed in PRP (Aoto et al., 2014), so the blood-drawing time was not unified.

This study has some limitations. First, only four preparers participated in PRP preparation. Because the amount of blood draws per donor increased, the number of preparers was limited. Second, the 17 samples were prepared on different days and the amount of time between preparations was not standardized, which might have affected assessment of the learning curve. Third, the amounts of PRP prepared for clinical use and for this study differed. The kit used in this study utilized 9–36 ml of peripheral blood, which allows the preparation of 2–8 ml of PRP. However, in many clinical cases, 8 ml of PRP was prepared. Therefore, each preparer prepared 4 ml of PRP, an amount lower than that used in clinical settings. Finally, with regard to our investigation of three factors and diurnal variation, the methods of optimizing PRP preparation using the PDGF-Endoret Kit has become clear, although other factors (such as the pH of PRP) can vary (Wahlstrom et al., 2007; Fitzpatrick et al., 2017). Further studies are needed to determine the effects of any other variable parameters.

## Conclusions

In this study, we showed that PRP samples prepared using the PRGF-Endoret system did not differ in quality, depending on the experience of the preparer, but standardized training in the procedure is recommended. Moreover, PRP preparation required standardization of the timing before the blood counts were measured to ensure consistent and accurate PRP quality. The results revealed that the growth factor concentrations in PRP differ depending on the method used for platelet activation. To accurately assess the clinical outcomes of PRP therapy, collection of basic data is essential. The current study provides useful, basic data for the appropriate application of PRP therapy.

## Abbreviations

CaCl_2_: Calcium chloride
HGF: Hepatocyte growth factor
IGF: Insulin-like growth factor
LP-PRP: Leukocyte-poor PRP
PDGF: Platelet-derived growth factor
PDGF-BB: Platelet-derived growth factor-BB
PPP: Platelet-poor plasma
PRP: Platelet-rich plasma
WBC: White blood cell
